# Mental Health Status among Married Working Women Residing in Bhubaneswar City, India: A Psychosocial Survey

**DOI:** 10.1155/2014/979827

**Published:** 2014-03-31

**Authors:** Ansuman Panigrahi, Aditya Prasad Padhy, Madhulita Panigrahi

**Affiliations:** Department of Community Medicine, Kalinga Institute of Medical Sciences, KIIT University, Campus-5, Bhubaneswar, Odisha 751024, India

## Abstract

Mental health is a major public health concern worldwide. This study aimed to assess the mental health status and its correlates among married working women residing in Bhubaneswar city of Odisha, India. A cross-sectional study was undertaken in 240 households involving 240 married working women following a multistage cluster random sampling design. Using the predesigned, pretested interview schedule and self-reporting questionnaire, all relevant information was collected. Our study revealed that 32.9% of study respondents had poor mental health and only about 10% of these women had sought any kind of mental health services. Logistic regression analysis showed that 3 predictors such as favourable attitude of colleagues, sharing their own problems with husband, and spending time for yoga/meditation/exercise had significant positive impact on the mental health status of married working women. A preventive program regarding various aspects of mental health for married working women at workplace as well as community level could be a useful strategy in reducing this public health problem.

## 1. Introduction

Mental health is one of the most important public health issues as it is a major contributor (14%) to the global burden of disease worldwide [1]. It means the ability to respond to diverse experiences of life with flexibility and a sense of purpose. It can be described as a state of balance between an individual and his surrounding world, a state of harmony between oneself and others [2]. Mental health is the foundation for well-being and effective functioning for an individual and for a community and that of women is important both for their own health and for the well-being of their children and families. Women are more likely than men to be adversely affected by mental disorders, the most common being anxiety and depressive disorders [3, 4].

Status of women in the society has been changing fast due to multiple factors such as urbanization, industrialization, increased level of education, awareness of rights, and media influence. More and more women prefer to be engaged in some kind of employment, so that they can contribute financially to their family. But the attitude towards women especially married women and their role in family has remained the same, as even today taking care of the family and children is considered as their primary responsibility. Thus carrying out duties and responsibilities both at home and workplace overstrains a married working woman, thereby leading to various psychological problems like role conflict, job strain, mental fatigue, stress, anxiety, frustration, depression, anger, phobias, and other social and emotional distress. All of these problems can interactively affect the mental well-being of working women and more so in married working women. Studies have shown that working women have poor mental health and higher level of depression compared to nonworking women [5]. Data on mental health among married working women in India is sparse and to the authors' knowledge there has been no such study conducted in the state of Odisha.

In this context, the present study was planned to be undertaken in Bhubaneswar city to assess the mental health status among married working women and their role perception and determine the mental health correlates, so that appropriate targets for interventions could be identified.

## 2. Materials and Methods

Bhubaneswar, the capital city of the state of Odisha, is situated at the intersection of 20°12′ N–20°25′ N latitude and 85°44′E–85°55′E longitude, 45 meters above sea level. It is located southwest of the rivers Mahanadi, Baitarani, and Brahmani on the western fringe of the mid-coastal plain. Bhubaneswar includes 60 wards with a population of about 8, 50,000.

The present cross-sectional study was undertaken in the selected nonslum areas during 2011-2012. Considering the prevalence of poor mental health among women of reproductive age group as 23% observed in an Indian study [6], precision as 10%, design effect as 3, and level of confidence at 95%, sample size was calculated as 204. Multistage cluster random sampling design was used to select the study population. Out of total 60 wards, 20% of wards, that is, 12 wards, were selected using simple random sampling technique. From each ward, one nonslum area was randomly selected and thus in total, 12 areas were chosen as clusters for the study purpose. In each selected study area, 20 households were recruited as study units using simple random sampling technique. Only married women in the age group of 20–49 years willing to participate were considered for the study purpose whereas nonvolunteers, pregnant women, and those having any known health disorder were excluded from the study. In case of presence of two or more study subjects in one household, only one respondent was randomly chosen and if no woman was found in the selected household, the next adjacent household was approached. Overall, 240 eligible willing women from 240 households were recruited as study subjects and interviewed after obtaining the informed consent keeping their identities anonymous. However, 24 women could not continue in the study till the end and thus finally 216 married working women were considered as study participants. Using the predesigned and pretested interview schedule, all relevant information regarding sociodemographic characteristics like age, education, occupation, income, addiction, and so forth, was collected. Also perception of women respondents regarding home situation, family, and social issues was assessed.

The self-reporting questionnaire (SRQ), a standardized instrument, was administered to measure the mental health status of the participating women. This is a 20-item questionnaire requiring yes/no responses and screens for the presence of anxiety and depressive disorders. The SRQ has been standardised in India in two separate studies [7, 8]. Patients who scored 7 or more on the SRQ were designated as having poor mental health and those with a score below 7 were designated as being normal. Women found to have poor mental health were counselled and referred to nearby health facility for further investigation and follow-up.

*Analyses.* Statistical procedures such as simple descriptive statistics (means, standard deviations, and proportions), measure of association like chi-square test, and binary logistic regression models were employed for the data analysis. The logistic regression coefficients (log odds) and exponential of these values, that is, the odds in favour of poor mental health, were derived from logistic regression model which represents the risk of outcome associated with category of independent variables. All analyses were performed with SPSS statistical software version 16.0.

## 3. Results

Table 1 depicts the mental health status of married working women. It was observed that nearly one-third of women had poor mental health outcome and only about 7 (9.8%) of them sought mental health services in public/private health care facilities during the last one year.

Mean age of the study population was found to be 34.02 ± 7.23 years. About three-fourths of the respondents were more than 30 years old. Out of 216 married working women, 175 (81%) belonged to nuclear family. More than half of women belonged to general caste and the rest were from other backward caste (OBC), scheduled caste (SC), and scheduled tribe (ST). Majority (84.2%) of study population had attained graduation and/or postgraduation. Almost half of the respondents were doing job in private organisation. Around 70% women had denied that their husbands were addicted to alcohol or any drug. In 77 households, monthly income was >rupees 15,000/- and 41 households had monthly income <rupees 5000/-. Considering parental status, 176 (81.5%) women had children. We observed that 16 (7.4%) women reported they had been exposed to psychological trauma during last year and 33 (15.3%) informed that any of her family members was chronically ill for at least one month during last 6 months. Bivariate analysis shows that younger age, addiction of husband to alcohol/drug, and presence of chronic illness among family members are significantly associated with poor mental health outcomes of married working women (Table 2).

It is evident from Table 3 that the primary reasons for taking up employment by married women were personal interest (45.4%) followed by financial constraint (38.2%) and husband's will (23.8%). Around two-thirds of women had started their career before marriage. Workplace atmosphere was conducive according to 152 (70.4%) respondents and more than 80% of women agreed that attitude of colleagues and husband/in-laws was favourable both at workplace and home, respectively. However, 94 (43.5%) study subjects told that their family members expected them to do the same work as nonworking women. Nearly 90% of women denied any kind of misunderstandings or quarrels with their husband over the use of their money and almost 85% women shared their own problems with their husband. Bivariate analysis shows that conducive atmosphere at workplace, favourable attitude of colleagues and husband/in-laws, and sharing their own problems with husband have significant positive impact on mental health outcomes of married working women.

Table 4 explores various family/social issues and their association with mental health outcomes. More than one-third of women experienced conflict between home and job responsibilities and almost 80% had the view that they were doing justice to both roles. 178 (82.4%) women said that they were participating in decision making process of family and almost the same number of women felt that there was change in attitude of contemporary men whereas nearly half of respondents had the belief in natural superiority of men. Further, majority (88.9%) of women opined that women's employment raised family/social status. About 60% of women had time to attend social obligation whereas almost 90% of women attended religious services more than once in a month. Approximately 80% of women did not spend any time for yoga/meditation/exercise. Bivariate analysis illustrates that women experiencing any home-job conflict, not participating in decision making process of family, and not spending time for attending social obligation and also for yoga/meditation/exercise had poor mental health outcome.

All the variables which were found to be associated with mental health outcome in bivariate analyses were entered into the logistic regression model to predict the odds of mental health outcomes among the women respondents (Table 5). It was observed that attitude of colleagues, sharing their own problems with husband, and spending time for yoga/meditation/exercise were significantly affecting the mental health status of married working women.

## 4. Discussion

In our study, we observed that the prevalence of poor mental health among married working women was 32.9% of which only about 10% of women with poor mental health had sought any kind of mental health services even though these services are available in various government and private hospitals and nursing homes including three private medical institutions established in Bhubaneswar city. This might be attributed to social stigma attached to mental disorders or lack of awareness about availability of such services. This finding highlights the urgent need for developing an effective preventive programme or improving the existing system emphasizing on provision of mental health services. Zeynep et al. found in their study that a considerable proportion (25.9%) of women were having any mental disorder and only 4.7% had ever received care from mental health services [9]. WHO also reported that the prevalence of mental disorders of women was 25% and majority of them did not seek any mental health service [10]. Adzlin et al. showed the prevalence of psychological distress among married working women as 22.8% [11]. Another study in Ethiopia showed the prevalence of mental distress in working women as 25.9% [12]. Bivariate analysis revealed that younger married working women had poor mental health as compared to older counterparts. This could be due to the reason that younger women might be starting to handle new additional responsibilities after marriage. Further, women whose husbands were addicted to alcohol or any drug and women who had family members suffering from chronic illness were found to be at increased risk of developing poor mental health. Various other studies also supported these findings [6, 12–14].

Home and workplace atmosphere played a major role in deciding the mental health status of married working women. Conducive workplace atmosphere, favourable attitude of colleagues at workplace, and favourable attitudes of husbands/in-laws at home were found to be protective of overall mental health. Kopp et al. established in their study that job related factors and social support from family were important predictors of mental health [15]. Women who were sharing their own problems with their husbands had maintained good mental health. In addition, we found that some family/social issues like those women who experienced job-family conflict or did not participate in decision making process of family were at increased risk of developing poor mental health whereas spending time to attend social obligations and devoting some time for yoga/meditation/exercise had good mental health outcome. Chandola et al. observed that both work-to-family and family-to-work conflict affected the mental health of men and women in three different countries [16].

Logistic regression model identified 3 important predictors of poor mental health outcome among married working women. We observed that women experiencing favourable attitude of their colleagues at workplace were about 4.5 times more likely to have normal mental health as compared to women who were facing an unfavourable or indifferent attitude of their colleagues. The likelihood of normal mental health rises about 4 times when women were sharing their problems with husbands versus women not doing so. Further, women who were not engaged in any yoga/medication/exercise had almost 7 times more chance of developing poor mental health as compared to women engaged in yoga/medication/exercise more than 2 times per week. These findings indicate that there should be sincere effort at workplaces in building healthy relationship among employees, thereby developing favourable attitude of colleagues towards each other which will help in maintaining good mental health, especially, of women employees. Also at family level, working women and their husbands must be counselled regarding the relevance of sharing their own problems with each other as it will not only help them find viable solutions for their problems but also prevent developing poor mental health. Community based orientation programs regarding various aspects of mental health should be organized at regular intervals for the working women emphasizing the beneficial effect of activities like yoga/mediation/exercise on mental health and thus they should be encouraged to incorporate such activities in their daily routine activities.

## 5. Limitation and Conclusion 

The cross-sectional design of our study precluded the ability to establish the casual association between the identified independent variables and poor mental health status. This study did not include married working women more than 50 years old which decreases the generalizability of study's findings to a wider section of older women. Again as information regarding various predictive variables was based on self-reports by respondents, it may lead to response bias. Despite these limitations, the results of the study had important practical implications such as assessment of the problem among married working women and identification of risk factors/predictors of poor mental health outcome which can help the policy makers and health professionals in planning essential intervention strategies to deal with this social stigma. Further analytic epidemiological research is needed to replicate the findings and find out potential predictive variables.

## Figures and Tables

**Table 1:** Mental health status of married working women (*N* = 216).

Variables	Number (%)
Mental health status	
Poor	71 (32.9)
Normal	145 (67.1)
Sought mental health services before
Yes	07 (9.8)
No	64 (91.2)

**Table 2:** Sociodemographic characteristics and bivariate associations with mental health outcomes among married working women.

Variables	Mental health status		
	Poor (%)	Normal (%)	*χ* ^2^, df, *P*
Age group (in years)			
20–30	26 (38.8)	41 (61.2)	**6.339, 2, 0.042**
31–40	35 (36.5)	61 (63.5)	
41–50	10 (18.9)	43 (81.1)	
Family type			
Joint	60 (34.3)	115 (65.7)	0.837, 1, 0.360
Nuclear	11 (26.8)	30 (73.2)	
Caste			
General	36 (31.6)	78 (68.4)	0.745, 2, 0.689
OBC	26 (36.6)	45 (63.4)	
SC/ST	09 (29.0)	22 (71.0)	
Educational status			
Intermediate	11 (32.4)	23 (67.6)	0.038, 2, 0.981
Graduate	43 (32.6)	89 (67.4)	
Postgraduate	17 (34.0)	33 (66.0)	
Occupational status			
Government	27 (29.0)	66 (71.0)	1.092, 2, 0.579
Semigovernment	06 (35.3)	11 (64.7)	
Private	38 (35.8)	68 (64.2)	
Addiction of husband to alcohol/drugs			
Yes	28 (43.1)	37 (56.9)	**4.390, 1, 0.036**
No	43 (28.5)	108 (71.5)	
Monthly income			
<Rs. 5,000	18 (43.9)	23 (56.1)	2.840, 2, 0.242
Rs. 5,000–15,000	29 (29.6)	69 (70.4)	
>Rs. 15,000	24 (31.2)	53 (68.8)	
Parental status			
Had child	55 (31.2)	121 (68.8)	1.131, 1, 0.288
Had no child	16 (40.0)	24 (60.0)	
History of previous trauma			
Yes	07 (43.8)	09 (56.2)	0.927, 1, 0.336
No	64 (32.0)	136 (68.0)	
Presence of chronic illness among family members			
Yes	16 (48.5)	17 (51.5)	**4.304, 1, 0.038**
No	55 (30.1)	128 (69.9)	

**Table 3:** Workplace and home situation and bivariate associations with mental health outcome.

Variables	Mental health status		
	Poor (%)	Normal (%)	*χ* ^2^, df, *P*
Reason for working			
Financial constraint	29 (38.2)	47 (61.8)	2.528, 2, 0.283
Personal interest	32 (32.7)	66 (67.3)	
Husband's will	10 (23.8)	32 (76.2)	
Started career			
Before marriage	50 (35.5)	91 (64.5)	1.235, 1, 0.266
After marriage	21 (28.0)	54 (72.0)	
Workplace atmosphere			
Conducive	40 (26.3)	112 (73.7)	**11.759, 2, 0.003**
Not conducive	18 (56.2)	14 (43.8)	
Neutral	13 (40.6)	19 (59.4)	
Attitude of colleagues			
Favourable	48 (26.5)	133 (73.5)	**20.419, 1, 0.000**
Unfavourable/indifferent	23 (65.7)	12 (34.3)	
Attitude of husband/in-laws			
Favourable	54 (29.8)	127 (70.2)	**4.666, 1, 0.031**
Unfavourable/indifferent	17 (48.6)	18 (51.4)	
Managing household affairs satisfactorily			
Almost always	09 (25.0)	27 (75.0)	3.507, 3, 0.319
Frequently	20 (35.1)	37 (64.9)	
Occasionally	28 (30.4)	64 (69.6)	
Rarely/never	14 (45.2)	17 (54.8)	
Family members expecting same work as nonworking women			
Yes	30 (31.9)	64 (68.1)	0.069, 1, 0.793
No	41 (33.6)	81 (66.4)	
Misunderstanding/quarrels with husband			
Yes	10 (41.7)	14 (58.3)	0.947, 1, 0.331
No	61 (31.8)	131 (68.2)	
Sharing own problems with husband			
Yes	55 (30.1)	128 (69.9)	**4.304, 1, 0.038**
No	16 (48.5)	17 (51.5)	

**Table 4:** Family/social issues and bivariate associations with mental health outcomes.

Variables	Mental health status		
	Poor (%)	Normal (%)	*χ* ^2^, df, *P*
Experiencing any conflict			
Yes	36 (42.9)	48 (57.1)	**6.213, 1, 0.013**
No	35 (26.5)	97 (73.5)	
Doing justice to both roles			
Yes	52 (30.4)	119 (69.6)	**2.253, 1, 0.133**
No	19 (42.2)	26 (57.8)	
Participation in decision making process of family			
Yes	53 (29.8)	125 (70.2)	**4.393, 1, 0.036**
No	18 (47.4)	20 (52.6)	
Change in attitude of contemporary men			
Yes	61 (33.7)	120 (66.3)	0.350, 1, 0.554
No	10 (28.6)	25 (71.4)	
Belief in natural superiority of men			
Yes	30 (30.0)	70 (70.0)	0.695, 1, 0.404
No	41 (35.3)	75 (64.7)	
Women's employment raising family/social status			
Yes	63 (32.8)	129 (67.2)	0.003, 1, 0.959
No	08 (33.8)	16 (66.7)	
Time for attending social obligation			
Yes	34 (26.6)	94 (73.4)	**5.665, 1, 0.017**
No	37 (42.0)	51 (58.0)	
Attending religious services			
0-1 time/month	62 (32.5)	129 (67.5)	0.125, 1, 0.723
>1 time/month	09 (36.0)	16 (64.0)	
Spending time for yoga/meditation/exercise			
Nil	63 (36.8)	108 (63.2)	**6.652, 2, 0.036**
1-2 times/week	06 (23.1)	20 (76.9)	
>2 times/week	02 (10.5)	17 (89.5)	

**Table 5:** Results of binary logistic regression predicting the odds of mental health outcomes among women respondents (*N* = 216).

Independent variable	Dependent variable		“*P*” value
	Mental health status (normal = 1, poor = 0)	
	(*β*)	Exp (*β*)
Age group (in years)			0.203
20–30	−0.816	0.442	0.104
31–40	−0.765	0.466	0.101
41–50^R^			
Addiction of husband to alcohol/drugs			
Yes	−0.690	0.502	0.061
No^R^			
Chronic illness of family members			
Yes	−0.201	0.818	0.691
No^R^			
Workplace atmosphere			0.152
Conducive	0.735	2.086	0.115
Not conducive	−0.054	0.948	0.933
Neutral^R^			
Attitude of colleagues			
Favourable	1.520	4.571	**0.004**
Unfavourable/indifferent^R^			
Attitude of husband/in-laws			
Favourable	0.809	2.245	0.086
Unfavourable/indifferent^R^			
Sharing their own problems with husband			
Yes	1.351	3.860	**0.005**
No^R^			
Experiencing any conflict			
Yes	−0.509	0.601	0.169
No^R^			
Time for social obligation			
Yes	0.661	1.937	0.063
No^R^			
Spending time for yoga/meditation/exercise			**0.019**
Nil	−1.932	0.145	**0.018**
1-2 times/week	0.867	0.420	0.365
>2 times/week^R^			
Participation in decision making process of family			
Yes	0.553	1.738	0.207
No^R^			

Note: R: reference category; *β*: regression coefficient (log odds ratio). Exp (*β*): odds ratio. The model fit statistics were as follows. Omnibus tests of model coefficients: *χ* ^2^ value of 62.342 with a “*P*” value of 0.000 tells that the final model as a whole fits significantly better than an empty model (model with no predictors). Hosmer and Lemeshow test: *χ* ^2^ value of 3.062 with a “*P*” value of 0.930 indicates that there is no significant difference between observed and predicted probabilities and thus the model fits.
